# Occurrence of Equine Foamy Virus Infection in Horses from Poland

**DOI:** 10.3390/v14091973

**Published:** 2022-09-06

**Authors:** Magdalena Materniak-Kornas, Wojciech Rożek, Jerzy Rola, Zbigniew Osiński, Martin Löchelt, Jacek Kuźmak

**Affiliations:** 1 Department of Biochemistry, National Veterinary Research Institute, 24-100 Pulawy, Poland; 2 Department of Virology, National Veterinary Research Institute, 24-100 Pulawy, Poland; 3 Department of Hygiene of Feeding Stuffs, National Veterinary Research Institute, 24-100 Pulawy, Poland; 4 Division of Viral Transformation Mechanisms, Research Program Infection, Inflammation and Cancer, German Cancer Research Center (DKFZ), 69120 Heidelberg, Germany

**Keywords:** equine foamy virus, horses, diagnostics, ELISA, PCR, prevalence

## Abstract

Equine foamy virus (EFVeca) is a foamy virus of non-primate origin and among the least-studied members of this retroviral subfamily. By sequence comparison, EFVeca shows the highest similarity to bovine foamy virus. In contrast to simian, bovine or feline foamy viruses, knowledge about the epidemiology of EFVeca is still limited. Since preliminary studies suggested EFVeca infections among horses in Poland, we aimed to expand the diagnostics of EFVeca infections by developing specific diagnostic tools and apply them to investigate its prevalence. An ELISA test based on recombinant EFVeca Gag protein was developed for serological investigation, while semi-nested PCR for the detection of EFVeca DNA was established. 248 DNA and serum samples from purebred horses, livestock and saddle horses, Hucul horses and semi-feral Polish primitive horses were analyzed in this study. ELISA was standardized, and cut off value, sensitivity and specificity of the test were calculated using Receiver Operating Characteristic and Bayesian estimation. Based on the calculated cut off, 135 horses were seropositive to EFVeca Gag protein, while EFVeca proviral DNA was detected in 85 animals. The rate of infected individuals varied among the horse groups studied; this is the first report confirming the existence of EFVeca infections in horses from Poland using virus-specific tools.

## 1. Introduction

Foamy viruses (FVs), also known as spumaviruses, are the least known complex retroviruses [1]. Some features of their replication pathway and complex genomic organization distinguish them from other retroviruses [2]. Infections with FVs are persistent with sustained and diagnostic antibody responses, mainly against the viral structural Gag protein but also against non-structural antigens, while the presence of viral DNA is not consistently detected, possibly due to the fact that target cells in infected animals are only in part known [2,3,4]. The most likely routes of FVs transmission are via transfer of blood, saliva and social interactions [2,5,6]. FVs infections are highly prevalent among several animal species including free-ranging and captive non-human primates (chimpanzees, gorillas, etc.), cats and cattle [2,7,8,9,10,11,12,13,14].

Equine foamy virus (EFVeca) [1] was first isolated by Tobaly-Tapiero and co-workers in 2000 from blood of a naturally infected horse [15], while another isolate was described almost twenty years later by Kirisawa et al. [16]. EFVeca genome organization is typical for foamy viruses, but its amino acid sequences show the highest similarity to bovine foamy virus (BFVbta), with over 45% homology between capsid Gag protein of EFVeca and BFVbta, while, EFVeca and BFVbta show only 24% and 23% homology to prototype FV (PFV) of simian origin, respectively. The first information on the seroprevalence of EFVeca infections in horses was published recently by Kirisawa et al. [16] who noted an average seroreactivity to EFVeca in 25% of 459 tested animals. In that report immunofluorescence assay was used for the detection of EFVeca in tested material, but at the moment, there is a lack of efficient and robust methods for the quick diagnostics of EFVeca infections in horses.

In general, foamy viruses are considered apathogenic, but some data suggest their role as co-factors in other viral infections, especially in the context of mixed infections [17] and some pathology in humans after zoonotic infections with simian foamy viruses (SFV) [18,19]. Previous reports suggested that co-infections with bovine foamy virus and bovine leukemia virus in cows, similar to co-infection with feline foamy virus (FFVfca) and feline immunodeficiency virus in cats, may impair the host immune defense capacity [20,21,22,23]. So far no data regarding possible EFVeca co-infections with other equine viruses have been reported.

The goal of our study was to investigate the prevalence of EFVeca infections in horses from Poland. In previous studies, Gag protein has been successfully utilized as diagnostic antigen in serological studies for all known foamy viruses [13,14,24,25], therefore, also in our study we developed EFVeca Gag-specific diagnostic ELISA and PCR tests and applied them to investigate the prevalence of EFVeca infections in horses from Poland. We also performed molecular analyses of newly-obtained EFVeca sequences representing Polish EFVeca field isolates and intended to determine the rate of co-infections of EFVeca with other common respiratory pathogens of horses like equine herpesvirus type 1 (EHV-1), equine arteritis virus (EAV) and equine influenza viruses (EIV).

## 2. Materials and Methods

### 2.1. Horse Samples

The samples submitted in this study were collected from 248 horses, 1–12 years old, including: 58 samples from 14 stables of Hucul horses (H), 25 the Konik horses, also called Polish primitive horses (K) from one stud farm (only 1–2 years old horses), 66 livestock horses (samples collected in three abattoirs) (D), 17 saddle horses from one stable (S) and 82 purebred horses from four stud farms (P) all over Poland. Blood samples were collected in the years 2012–2013 from the jugular vein to the tubes with anticoagulant (EDTA disodium salt) and for serum preparation. Samples with anticoagulant were centrifuged at 1500× *g* for 30 min at room temperature and the buffy coat was collected and subjected to osmotic hemolysis using ice cold water and 4.5% NaCl. Peripheral blood leukocytes (PBLs) were recovered by centrifugation at 800× *g* for 10 min, washed twice in PBS and aliquoted for storage at −70 °C as dry pellet until DNA extraction. Blood samples without anticoagulant were centrifuged at 1500× *g* for 30 min at room temperature, and obtained serum samples were collected for serological testing and stored frozen at −20 °C until assayed.

### 2.2. DNA Preparation

Total DNA was extracted from peripheral blood leukocytes using DNeasy Blood and Tissue Kit (Qiagen, Hilden, Germany) following the manufacturer’s instructions and stored at −20 °C until use. DNA concentrations were measured spectrophotometrically using NanoPhotometer (Implen, München, Germany).

### 2.3. EFVeca DNA Detection

Semi-nested PCR was performed using one microgram of genomic DNA from blood cells using a set of external primers: EFVpolF1: TGGGAAAATCAAGTGGGACATA, EFVpolR: TACAACATCTGCAGTAAATAAGGC and EFVpolF2: ATGGACGATGGAGAATGGTACTGGAT (Genomed, Warsaw, Poland) with EFVpolR primer as internal primers, which were designed to match the corresponding part of the *pol* gene encoding for reverse transcriptase of EFVeca (GenBank NC_002201.1 and LC381046.1). First amplification included 2.5 U of OpitTaq DNA polymerase (EURx, Gdansk, Poland), 1× PCR buffer with 1.5 mM MgCl_2_, 0.2 µM of each primer, 0.4 mM of dNTP-mix (ThermoScientific, Waltham, MA, USA) and 1 µg of genomic DNA. The cycling parameters consisted of initial denaturation at 94 °C for 3 min, followed by 35 cycles of denaturation at 94 °C for 45 s, annealing at 54 °C for 45 s and elongation at 72 °C for 1 min, with final elongation at 72 °C for 5 min. Semi-nested amplification was done at similar conditions, except for annealing temperature set at 60 °C, using 1/10 volume of first PCR as a template. The expected size of amplicon was 275 bp. The resulting amplicons were analyzed on 1% agarose gels, and nineteen of them were cloned into the pCR2.1-TOPO vector (Invitrogen, Carlsbad, CA, USA), and sequenced in both directions according to the method of Sanger [26] by Genomed (Warsaw, Poland) using a 3730xlDNA Analyzer (Applied Biosystems, Foster City, CA, USA) and a BigDye Terminator v3.1 Cycle Sequencing Kit.

### 2.4. EFV Sequence Analysis

In order to perform a phylogenetic analysis of 19 cloned sequences encoding a fragment of EFVeca *pol* gene, the sequences were devoid of primers and aligned to corresponding sequences derived from complete genomes of two available EFVeca isolates and with other non-primate FVs available in the GenBank, using the Geneious alignment module within Geneious Pro 5.3 software (Biomatters Ltd., Auckland, New Zealand). The alignment was submitted to the MEGA 6 version [27] for the best model selection measured by the Bayesian information criterion (BIC) and the corrected Akaike information criterion (AICc). According to the results of the Tamura 3-parameter substitution model with gamma distribution (+G) with five rate categories [28] were applied in MEGA 6 to infer a phylogenetic tree using the maximum likelihood method. The statistical confidence limits of the phylogram topologies were assessed with 1000 bootstrap replicates [29].

### 2.5. EFVeca Gag Amplification and Selection of a Sequence for Recombinant Antigen Preparation

The full-length *gag* gene of EFVeca was amplified from leukocytes of six horses positive in semi-nested PCR (Section 2.3) with primers designed to match EFVeca isolates available in GenBank (NC_002201.1 and LC381046.1). The first PCR included external primers Ngag5: ACCTGGGCTACTTAAGAGA and Ngag3: TATTACCTCAGCTGCTAA, while nested PCR internal primers Gag5: GGATCCATGGCTCAAAACGAGACT and Gag3: CTCGAGTTAAGGCTGTTGCCCTTGA. The first PCR was performed in a TGradient thermocycler (Biometra, Göttingen, Germany) under the following conditions: 94 °C for 3 min and 35 cycles of 94 °C for 45 s, 55 °C for 45 s and 72 °C for 1.5 min, with additional final elongation step of 72 °C for 10 min. The PCR reaction mixture consisted of 1 U Phusion Hot Start II DNA Polymerase (ThermoScientific, Carlsbad, CA, USA), 1× PCR buffer, 2.5 mM MgCl_2_, 0.15 mM of each dNTP and 0.4 μM of each primer. 0.5 μg of genomic DNA was used as the template. Nested amplification was done at similar conditions, except for annealing temperature set at 60 °C, using 1/10 volume of first PCR as template. The expected size of the amplicon was 1692 bp. The nested PCR product obtained was subcloned into pDRIVE vector (Qiagen, Hilden, Germany) and clones were sequenced by Sanger method in both directions (Genomed, Warsaw Poland). The obtained full-length *gag* consensus sequences from six horses were translated into amino acid sequences and aligned with EFVeca references available in GenBank using the Geneious alignment module within Geneious Pro 5.3 software (Biomatters Ltd., Auckland, New Zealand).

Additionally, Geneious Pro 5.3 software (antigenic plug-in) was used to predict potentially antigenic regions of a protein sequence, using the method of Kolaskar and Tongaonkar [30,31], which bases on the analysis of data from experimentally determined antigenic sites on proteins which revealed that the hydrophobic residues cysteine (C), leucine (L) and valine (V) are more likely to be a part of antigenic sites if they are present on the surface of a protein. The method of Kolaskar and Tongaonkar to predict antigenic determinants in proteins is semi-empirical and makes use of physicochemical properties of amino acid residues and their frequencies of occurrence in experimentally known segmental epitopes and the calculated score reflects the strength of prediction.

For further recombinant protein development, one sequence with the highest homology to reference isolates was chosen.

### 2.6. Development of Recombinant Gag Protein of EFVeca

The specific primers with adaptors (pLateGag5: GGTTGGGAATTGCAAATGGCTCAAAACGAGACT; pLateGag3: GGAGATGGGAAGTCATTAGGGTCGACCAAGAGGATCA) were used to amplify a 1225 bp fragment of EFVeca *gag* gene from horse no. 58-9; this fragment of EFVeca *gag* gene encodes a 385 aa of N-terminal/central part of EFVeca Gag protein (EFV Gag aa 1 to 385). The PCR reaction was performed in TAdvanced thermocycler (Analytic Jena, Göttingen, Germany) under the following conditions: 98 °C for 30 s and 30 cycles of 98 °C for 10 s, 72 °C for 30 s, with an additional final polymerization step of 72 °C for 10 min. PCR reaction mixture consisted of 1 U Phusion Hot Start II DNA Polymerase (ThermoScientific, Carlsbad, CA, USA), 1× PCR buffer with 1.5 mM MgCl_2_, 0.2 mM of each dNTP and 0.5 μM of each primer. The full-length *gag* clone (100 ng) derived from horse no. 58-9 was used as the template. The amplicon of the expected size was extracted from the agarose gel (NucleoSpin Gel and PCR Clean-up Mini kit; Macherey Nagel, Düren, Germany) and was fused in frame with His-tag in pLate52 vector using the ligation independent aLICator Cloning and Expression System (GE Healthcare, Chicago, IL, USA). *E. coli* Rosetta (DE3) pLys cells were transformed with the obtained construct, and positive clones were identified by PCR described above and sequenced (Genomed, Warsaw, Poland). For fusion protein expression, colonies of *E. coli* Rosetta cells transformed with the LATE52/gag plasmid were grown at 37°C in LB medium containing 100 μg/mL ampicillin. At 0.7 OD_600nm_, recombinant protein expression was induced by adding 1 mM isopropyl-D-thio-galactoside (IPTG, Sigma-Aldrich, St. Louis, MO, USA) to the medium and bacteria were incubated for another 5 h at 37 °C and harvested by centrifugation at 4000 rpm. His-tagged recombinant Gag protein was purified from the bacterial lysate using the TALON Metal Affinity Resin kit under denaturing conditions (Clontech, Mountain View, CA, USA). Pelleted bacteria were resuspended in binding buffer (50 mM sodium phosphate pH 7.0, 300 mM NaCl, 5 mM imidazole, 1 mM β-mercaptoethanol, 8 M urea) followed by incubation at room temperature (RT) for 20 min with gentle agitation and disrupted by sonication (3 pulses of 5 µ, 30 s each on ice with 1 min intervals). The cell lysate was centrifuged at 11,000× *g* for 15 min at 4 °C and the obtained supernatant was passed through a 0.45 µm filter (Millipore, Merck, Darmstadt, Germany) and incubated with equilibrated TALON resin for 30 min at room temperature with agitation. Beads were loaded into a 2 mL column, and washed two times with the same buffer. His-tagged Gag protein was eluted with elution buffer (similar to binding buffer except the addition of 150 mM imidazole). The eluate was dialyzed against PBS overnight at RT. Purified protein was analyzed by SDS-PAGE, aliquoted and stored at −20 °C until it was used as antigen in immunoblotting and ELISA.

### 2.7. Immunoblotting for the Detection of the Horse Sera Reactivity to EFVeca Recombinant Antigen

20 µg of purified recombinant EFVeca Gag protein was separated by SDS-PAGE and served as antigen for western blotting analyses (WB) [32]. Horse serum samples and controls were used at 1:100 dilutions (*v/v* in 2% bovine albumin, 0.01% Tween 20, PBS) and Protein G-peroxidase conjugate (Sigma-Aldrich, St. Louis, MO, USA) at 1:10,000 dilution. 4-chloro-naphtol (Sigma-Aldrich, St. Louis, MO, USA) was used as the substrate for peroxidase. Immunoblotting was established and optimized using 29 horse sera from semi-nested PCR-positive horses and 40 serum samples from PCR-negative individuals.

### 2.8. EFVeca Gag Specific ELISA Test

ELISA was used to examine antibody response to EFVeca Gag protein in the sera of horses. In brief, 96-well microtiter plates (Thermo Labsystems, Dreieich, Germany) were coated with Gag recombinant protein in carbonate buffer at a concentration of 7 μg/mL, blocked with 0.2% (*w/v*) casein and 0.05% (*v/v*) Tween 20 in PBS (blocking buffer). All sera were incubated at a dilution of 1:100 in blocking buffer containing 0.2% of casein from bovine milk (Sigma-Aldrich, St. Louis, MO, USA) and were incubated for 1 h at RT in the coated ELISA plate wells, washed, and incubated for 45 min at RT with Protein G-peroxidase conjugate at 1:10,000 dilution (Sigma-Aldrich, St. Louis, MO, USA). TMB (Sigma-Aldrich, St. Louis, MO, USA) was added as substrate. Pools of three serum samples from naturally EFVeca infected horses and three from uninfected animals, previously diagnosed by PCR and western blotting test with recombinant antigen, were used at 1:100 dilutions, as the positive and negative control, respectively. Each sample and controls were tested in duplicate. Optical density (OD) measurements were done at a wavelength of 450 nm and the results were expressed as a sample-to-positive ratio (S/P-ratio = (OD sample-OD negative control)/(OD positive control-OD negative control). To assess the cut-off value for positive sera a Receiver Operating Characteristic (ROC) analysis was performed, using MedCalc for Windows, version 20.014 (MedCalc Software, Ostend, Belgium). Evaluation was performed by testing of 111 horse sera of known serological status determined by immunoblot; these sera included 65 positive and 46 negative samples. Additionally, the sensitivity and specificity of newly developed ELISA were estimated using Bayesian analysis. The results from both, ELISA and immunoblot were used for estimation of the prior distributions, which were specified by modelling to generate the posterior distribution probabilities of sensitivity and specificity.

### 2.9. Detection of Antibodies Specific for EHV-1, EAV and EIV

The presence of antibodies specific for equine herpesvirus type 1 (EHV-1) and equine arteritis virus (EAV), were tested by sero-neutralisation tests, while the hemagglutination inhibition (HI) test was performed to screen for antibodies against equine influenza viruses subtype 1 and 2 (EIV-1 H7N7 and EIV-2 H3N8). All tests were done according to the standards of the World Organisation for Animal Health (WOAH) procedures described in the Manual of Diagnostic Tests and Vaccines for Terrestrial Animals [33,34,35] and are routinely used for diagnostics in the Department of Virology at NVRI.

### 2.10. Statistical Analysis

ROC calculation and generation of graphs were done using MedCalc for Windows, version 20.014 (MedCalc Software, Ostend, Belgium).

Bayesian modelling in WinBUGS software was performed to estimate the diagnostic sensitivity and specificity of the newly developed ELISA test. The two-test, one population scenario, which incorporates the co-variances between tests was used [36]. To compare the performances of the two methods, the McNemar’s chi-square test was used.

Pearson’s chi-square was used to check whether there is an association between the seroreactivity to EFVeca Gag protein and the infection with other equine viruses. Calculations were done using MedCalc for Windows, version 20.014 (MedCalc Software, Ostend, Belgium).

## 3. Results

### 3.1. Detection of EFVeca DNA in Peripheral Blood Leukocytes of Horses

EFVeca DNA was detected in PBLs using semi–nested PCR with primers flanking a 275 bp fragment of the conservative region of *pol* gene encoding reverse transcriptase. The positive reference sample was a plasmid carrying EFVeca *gag* and *pol* genes (generously provided by Dr. A. Saib and Dr. Joelle Tobaly-Tapiero). The specific amplification product was obtained for 85 horses (34.3%) out of 248 tested. The PCR specificity was confirmed by sequencing of semi-nested PCR products derived from the leukocytes of 19 horses.

The comparison of those 19 nucleotide sequences with the corresponding reference EFVeca sequences showed that the overall homology ranged between 92.3–97.1% to Japanese isolate LC381046 and 95.7–100% to French isolate NC_002201.1.

Interestingly, although the fragment chosen for amplification was placed in the most conservative region of *pol* gene, some substitutions were noted in the sequences derived from Polish horses. Most of the observed variations related to C/T and G/A substitutions, which led to a nonsense mutation in the sequence derived from Hucul horse 316-6H (G to A at positions 139 and 157); these types of mutations are known to be APOBEC-induced changes [37]. Additionally, the deletion at position 12 generated stop codons in sequences of four horses, including two Hucul horses (316-11H, 301-13H) one livestock (308-21D) and one saddle horse (315-7S), while insertions of a G residue at position 92 and 95 in the sequence from Hucul horse no. 316-8H led to a frame-shift changing the amino acid sequence.

**Figure 1:**
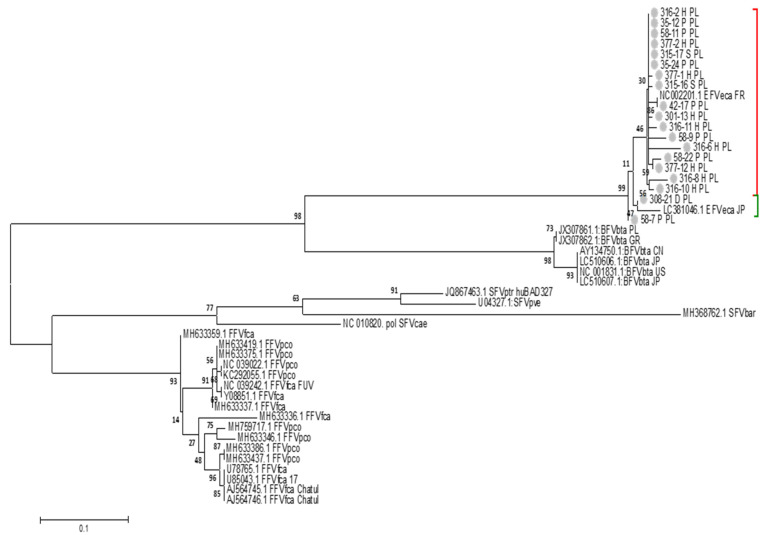
Phylogenetic tree inferred from the 208 bp sequence of the EFVeca *pol* region amplified from Polish horses (PL) and extracted from sequences of FVs isolates available in GenBank (EFVeca, BFVbta, feline foamy viruses: FFVfca and FFVpco, simian foamy viruses: SFVpve, SFVptr_huBAD327, SFVbar, SFVcae). All analyzed sequences were devoid of primer sequences. The analyses were conducted in MEGA 6 using the Maximum Likelihood method and Tamura 3-parameter model with the gamma distribution (+G) with five rate categories. Numbers at the branches indicate the percentage of bootstrap values obtained from 1000 replicates. Grey nodes correspond to EFVeca sequences obtained in this work; red bracket corresponds to the sequences clustering with French EFVeca isolate, green bracket corresponds to the sequences clustering with Japanese EFVeca isolate.

The phylogenetic analysis showed that 17 sequences derived from: Hucul horses (316-11H, 316-6H, 316-2H, 316-8H, 301-13H, 316-10H, 377-1H, 377-2H and 377-12H), saddle horses (315-17S, 315-16S and purebred horses (42-17P, 58-9P, 58-22P, 58-11P, 35-24P, 35-12P) clustered together with prototypic European EFVeca isolate from France (NC_002201.1_EFVeca_FR) (Figure 1). Only one sequence, 308-21D, derived from a Polish livestock horse, clustered together with the reference sequence of the Japanese isolate of EFVeca (LC381046.1_EFVeca_JP), however with the low bootstrap support. Interestingly, one sequence from purebred horse 58-7P clustered separately from European as well as Japanese isolate, however, this sequence shared a unique motif of TTTA at nucleotide positions 200–203 (data not shown) with Japanese reference isolate and sequence from livestock horse 308-21D.

### 3.2. Analysis of EFVeca Gag Sequences Derived from Horses

**Figure 2:**
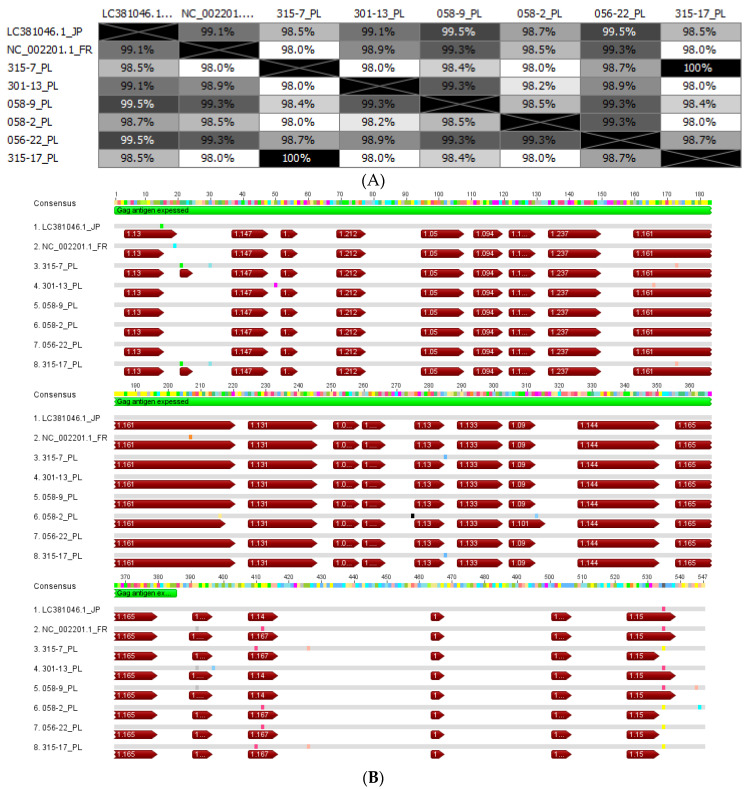
Homology between Gag amino acid sequences derived from EFVeca isolates available in GenBank and naturally infected horses from this work, sequences were devoid of primers: (**A**) pairwise percent identity of Gag amino acid sequences, the intensity of the color is correlated with pairwise percent identity; (**B**) alignment of Gag protein sequences with predicted antigenic regions indicated by red arrows with numbers reflecting the scores of prediction. Green bar corresponds to the N-terminal/central fragment of Gag protein used as antigen in serological tests. Amino acid changes are indicated, a nonsense codon is indicated in black.

The full-length *gag* gene of EFVeca was amplified from leukocytes of six horses determined as EFVeca positive in the semi-nested PCRs described above. The EFV *gag* amplicons were cloned, sequenced and aligned to reference sequences of EFVeca (NC_002201.1 and LC381046.1). The bioinformatic analysis of deduced EFVeca Gag amino acid sequences showed an 98–99.5% pairwise identity between sequences derived from horses and reference isolates of EFVeca (Figure 2A). Among analyzed sequences only one showed the presence of a single nonsense mutation (58-2) (Figure 2B). The sequence derived from horse No. 58-9 showed the highest homology to all sequences (98–99.5%) including references (99.3% to NC_002201.1 and 99.5% to LC381046.1).

In order to check whether the observed mutations influenced the seroreactivity of deduced Gag proteins, antigenic domains within EFVeca Gag protein were predicted (Figure 2B). The positions of deduced B cell epitopes appeared to be very consistent between the different EFVeca isolates and the reference isolates. Most of them were located in the matrix and capsid domains of Gag protein [38]. Only some mutations in animals no. 315-7, 315-17, 58-2 resulted in the change of amino acid which slightly altered the antigenic pattern of the Gag protein (Figure 2B).

### 3.3. Development and Optimization of EFVeca Specific ELISA

Based on the results of the Gag amino acid sequence comparison, a 385 aa N-terminal/central fragment of EFVeca Gag (Figure 2) of horse 58-9 was chosen to be expressed as the recombinant protein. The selected Gag fragment encompasses most of the putative and highly conserved antigenic sites of Gag being a good candidate for an antigen in the diagnosis of EFVeca infections.

**Figure 3:**
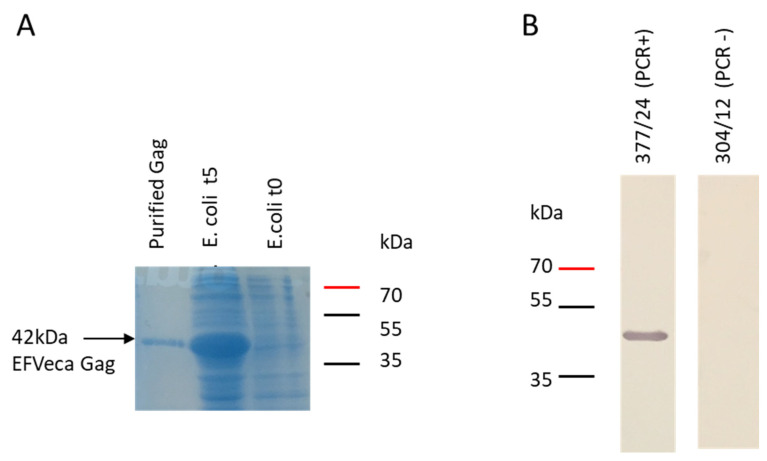
Confirmation of the overexpression of N-terminal/central part of EFVeca Gag protein; (**A**) SDS-PAGE with the *E.coli* lysate before (t0) and five hours after induction with IPTG (t5) and purified Gag protein; (**B**) detection of EFVeca Gag antigen by immunoblotting with horse serum samples from PCR EFVeca positive and negative individuals. The band of approximately 42 kDa corresponds to the recombinant EFVeca Gag antigen fused with His-tag.

Briefly, the partial *gag* gene of EFVeca was amplified from the full-length clone of the *gag* gene derived from PCR-positive horse 58-9 and cloned into the pLATE52 expression vector (ThermoScientific, Carlsbad, CA, US) with an N-terminal 6×His-tag. The overexpression of recombinant Gag protein was performed in *E.coli* Rosetta strain and was induced by 1 mM IPTG. The presence of desired EFVeca Gag was confirmed by SDS-PAGE (Figure 3A). As expected, the EFVeca Gag recombinant protein had a molecular size of approximately 42 kDa. The antigen was purified from the bacterial lysates by affinity chromatography under denaturing conditions through a 6×His tag flanked at their N-terminus/central Gag, and its seroreactivity was confirmed by immunoblotting (Figure 3B).

**Figure 4:**
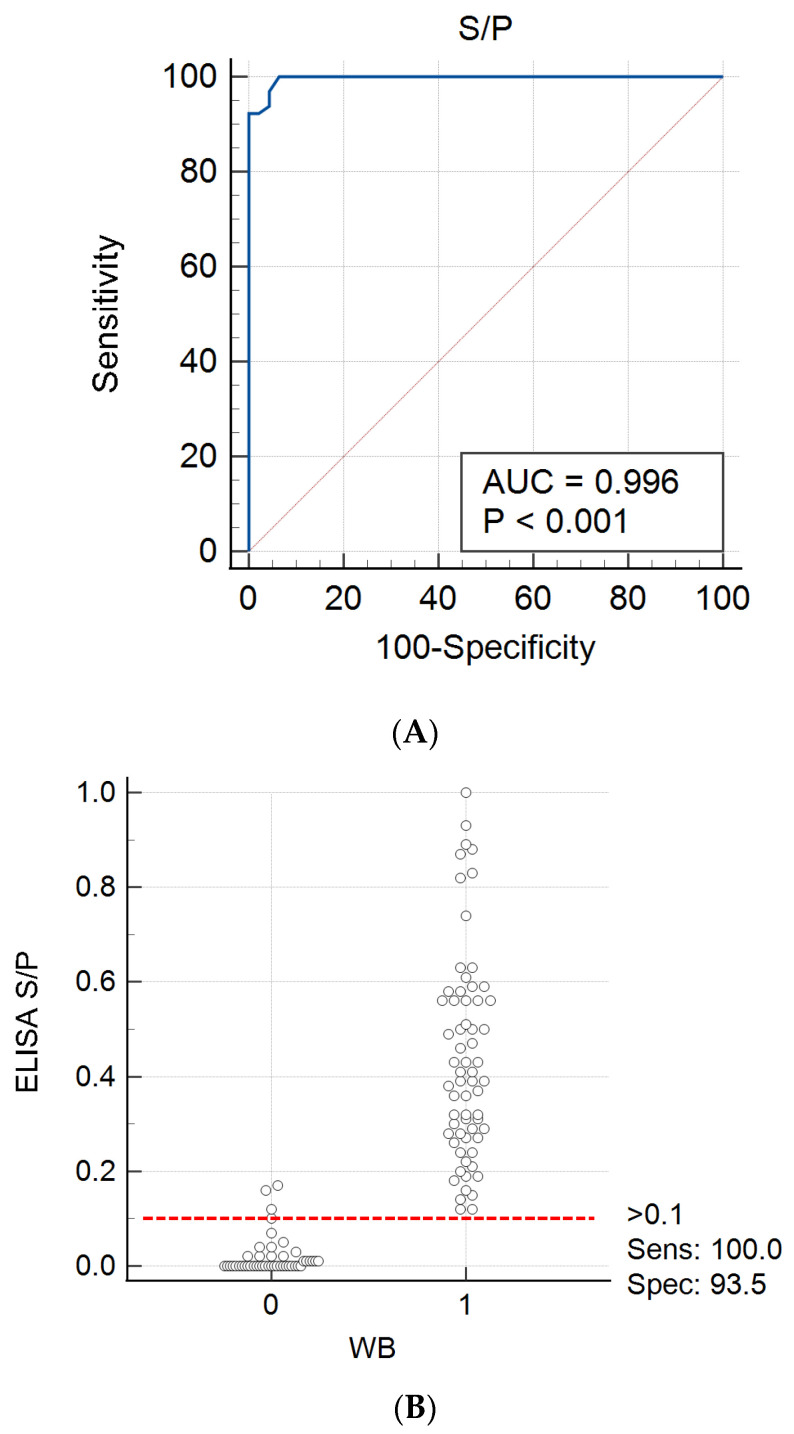
ROC curve (**A**) and the distribution of serological reactivity of horse samples to EFVeca Gag in ELISA and immunoblotting (WB) (**B**). Each grey circle represents S/P value of an individual serum.; numbers on X-axis refer to serum samples negative (0) and positive (1) in immunoblotting; red dashed line indicates the ELISA cut-off value for positive sera (S/P > 0.1). Commas were used as decimal points due to software settings.

Then, the purified antigen was used in ELISA and the test conditions were optimized using negative and positive control sera, previously determined by immunoblotting as described in Section 2.8. In order to establish a cut-off value for positive and negative sera, 111 serum samples with known EFVeca serological status were tested by ELISA, the signal to positive ratios (S/P) were calculated and a single-graph ROC plot was generated (Figure 4). Samples were judged to be positive if the S/P ratio was higher than 0.1. Under this condition, three sera, out of 45 negative sera as determined by immunoblot using the recombinant EFVeca Gag (data not shown) were considered positive by ELISA, two of them with S/P of 0.17 and 0.12 were also PCR negative and one (S/P of 0.16) was PCR positive. All 65 sera recognized as positive by immunoblot were also determined as positive by ELISA. Using standard formula, the sensitivity and specificity of the ELISA were 100% and 93.5%, respectively. The value of the area under the curve (AUC) 0.996 indicated that the ELISA was also highly accurate.

Additionally, a statistical analysis using Bayesian modelling was performed using the two-test, one population scenario. Prior distributions for sensitivity and specificity of the ELISA and WB were known from ROC analysis and the available literature [39]. The posterior information confirmed high sensitivity and specificity of the newly developed ELISA (Table 1). The differences observed between tests were not statistically significant, as was confirmed by McNemar’s chi-square test (chi = 1.333, *p*-value = 0.2482).

### 3.4. Serological Testing of Horse Samples Using a Newly-Developed EFVeca ELISA

The presence of antibodies specific to EFVeca Gag was tested by ELISA in total in 248 horse serum samples which included: 58 samples from Hucul horses, 25 from the Konik (Polish primitive horses), 66 from livestock horses, 17 from saddle horses and 82 purebred horses; this population of samples included also sera previously used for validation procedure (Section 3.3).

**Figure 5:**
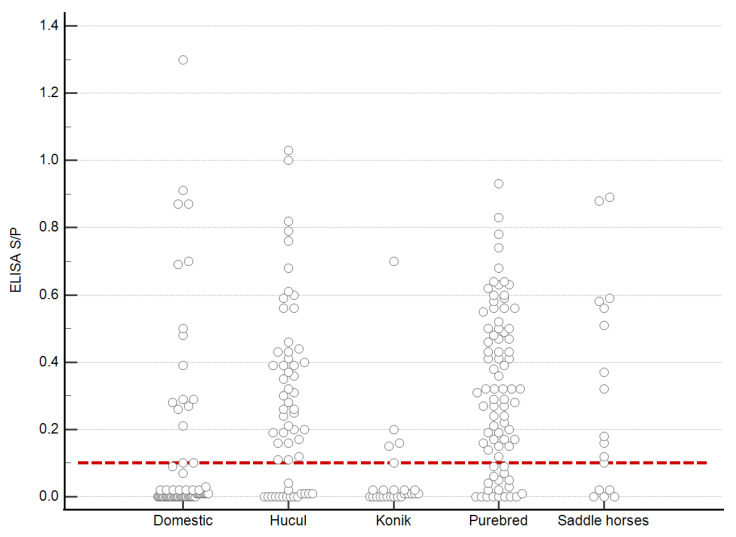
Distribution of EFVeca Gag reactivity in serum samples from different horse breeds. Each grey circle represents the S/P of an individual serum, dashed red line indicates the cut-off value of ELISA (S/P > 0.1). Commas were used as decimal points due to software settings.

The highest number of EFVeca seropositive samples was noted in the group of Hucul horses (72.4%) and purebred horses (76.8%) (Figure 5, Table 2). In the group of saddle horses over 60% of samples clearly showed the presence of EFVeca-specific antibodies. The lowest number of seropositive samples was observed in the group of livestock horses and the Konik, 21.2% and 16%, respectively (Table 2).

Interestingly, among the 85 horses recognized as PCR positive, 76 (89.4%) were also seropositive, while in sera of the remaining 9 PCR-positive individuals, EFVeca-specific antibodies were not detected. In the group of the 163 semi-nested-PCR negative horses, 104 (63.4%) were also seronegative, while 59 showed the presence of specific antibodies in ELISA (Table 2 and Table 3).

### 3.5. Co-Infections of EFVeca and Other Common Equine Viruses

A group of 81 serum samples diagnosed as seropositive and seronegative to EFVeca Gag (60 positive and 21 negative animals) were tested for the presence of antibodies against other equine viruses responsible for the most common respiratory infections in horses including: equine herpesvirus type 1 (EHV-1), equine arteritis virus (EAV), equine influenza viruses subtype 1 and 2 (EIV A1 H7N7 and EIV A2 H3N8). When the relationship between EFVeca and EIV A1/A2, EHV-1 and EAV infections was tested, statistically significant association was found only between EFVeca and EAV infections (*p* = 0.0003) (Table 4).

## 4. Discussion

Infections with known FVs are highly prevalent in their natural hosts. For instance, the seroprevalence of simian foamy virus (SFV) among primates varies between 24–100% in different primate species and under different conditions [11,40,41,42], while for other foamy viruses like feline foamy virus (FFVfca) in cats or BFVbta in cattle, seroprevalence value vary from 30 to 100% and from 7 to 50%, respectively [13,39,43,44]. A recent report showed that the seroprevalence of EFVeca in Japanese horses reaches 25%, which was confirmed in a study involving over 400 horses from several stud farms [16]. Currently, for nearly every exogenous FV species, serological tests are available, including immunofluorescence, immunoblotting and ELISA-based assays. Most of the FV-specific immunological assays developed so far are based on the reactivity against the Gag proteins, which was proved to be the antigen of choice for FVs serological studies [13,14]. That was confirmed by many studies showing that reactivity towards Gag, either in ELISA or western blots, has high diagnostic value for FV infections in primates [25,45,46], cats [14,47] and cattle [13,39]. PCR has also been used as a diagnostic method; however, it is considered as less sensitive due to the foamy virus latency in the course of infection, very low viral loads in different tissues and not fully recognized target tissues and replication sites [3,25,48,49].

In our study we, however, began with the identification of EFVeca-positive horses using semi-nested PCR. Our PCR results showed the presence of EFVeca DNA in almost 35% (85/248) of tested horses. The identification of horses with confirmed presence of EFVeca DNA was a key element for this study as it allowed molecular and phylogenetic analysis of the Polish EFVeca isolates and made it possible to identify infected and uninfected horses. During this study, no EFVeca reference serum samples were available, therefore a panel of sera taken from infected and uninfected horses was established based on the results of the PCR analysis, a method generally considered to have a lower sensitivity for FV diagnosis due to the fact that true target cells are not fully defined and their low abundance in naturally infected animals. The phylogenetic analysis of randomly selected amplicons from 19 horses showed that one sequence derived from purebred horse was almost identical to the European isolate of EFVeca, while 16 additional isolates were very closely related to this reference sequence. Interestingly, one sequence, 308-21D derived from a livestock horse, clustered with Japanese isolate of EFVeca but with poor bootstrap support, while one from purebred horse 58-7P clustered separately, however shared unique motif with Japanese isolate and 308-21D sequence. Several sequences amplified from Hucul horses clustered together, despite that the donor horses came from different stables. Considering that the collected and analyzed sequences came from a very conserved fragment of the EFVeca *pol* gene, we can assume that, similarly to BFVbta [50], different EFVeca clades have different geographic distributions or display breed-specific circulation pattern. The sequence analysis also revealed that six analyzed sequences underwent nonsense mutations due to deletions or G to A substitutions, which corresponds to antiviral activity of host antiviral APOBEC3 editing [37].

The results of the PCR screening test allowed us to select several EFVeca PCR positive horses to analyze the sequences of full-length of EFVeca Gag. The homology between the amino acid sequences of Gag from different horses and reference strains was very high and reached even 99.5%. Additional analysis of antigenic sites confirmed very conserved patterns of epitopes in most of the sequences; this, in fact, indicates that the changes present in the in-house amplified and expressed Gag protein did not alter the recognition of specific antibodies induced by natural EFVeca infection. For further development of ELISA with recombinant Gag protein as diagnostic antigen, animal/isolate 58-9 with homology scores of 99.3–99.5% to the other known EFVeca isolates (NC_002201.1 and LC381046.1) was chosen as representative antigen. A N-terminal/central domain of this Gag variant was highly expressed in induced bacteria, easily purified by His-tag—metal affinity chromatography and successfully used as antigen to screen horse sera for the presence of EFVeca-specific antibodies.

Our newly developed ELISA test, like all home-made ELISA assays, required validation to correctly identify serologically positive animals and to reduce the possibility of their misclassification. Although the ELISA is the most used technique for estimation of seroprevalence of foamy viruses [13,14,39,51] (and most other infectious agents), several studies documented that the use of immunoblot is an often more reliable technique to either confirm or deny the results obtained by ELISA [39,52,53]. We used the distribution of immunoblot results as a reference status estimation for ELISA cut-off calculation. When ROC analysis was performed, a cut-off S/P of 0.10 gave the optimum sensitivity of 100% and specificity of 93.5%; this relatively lower specificity was due to the fact that three sera, previously identified as negative by the immunoblot, showed a positive result in the ELISA; it can be assumed that a false-positive reaction is possible since recombinant Gag protein purified by a single-step extraction and enrichment method likely still contains bacterial or other antigens reacting with inherent factors present in some but not all serum samples mimicking non-specific reaction against the antigen. On the other hand, immunoblot is subjected to some level of uncertainty caused by subjective interpretation of test results, especially weak positive results, and the use of the same antigen preparation, like was used in ELISA. Therefore, the Bayesian estimation was applied since this technique is especially useful for estimation of diagnostic test performances in the absence of the gold standard method [36]. Our data showed that posteriori parameters, calculated by Bayesian estimation, were 97.3% and 91.1% for sensitivity and specificity, respectively; these values were somewhat lower from those calculated by ROC analysis (apparent parameters), however, the differences between tests were not statistically significant, as was showed by McNemar’s chi-square test (chi = 1.333, *p*-value = 0.2482).

Next, when ELISA was used to estimate the seroreactivity to EFVeca Gag, 55.6% of tested horse sera showed specific antibodies. Compared to other studies on FV-specific seroprevalence [13,14,39] our analysis showed a relatively high percentage of seropositive individuals, but their distribution varied in different groups of horses, representing different breeds. The reasons for such differences may be due to different breeding and animal husbandry practices or simply the age of the animals tested. As proven for other FVs, the risk of viral infection increases with the age and the number of animals living in the same herd or group [44,54] as was recently reported also for EFVeca [16]. Such a correlation can be observed also in our study: the age group of about one to two-years-old the Konik horses showed the lowest seroprevalence (16%) while animals over 10 years in the Hucul group and purebred horses had an over 70% seroprevalence (Table 2). Currently, there is no information available concerning the route (s) of EFVeca transmission, but it is likely that it is similar to that of BFVbta, which is suggested to be transmitted by saliva, through shared grazing areas or direct social or environmental contacts [5,6]; this can be especially important for the Hucul horses in semi-free ranging conditions which fosters the typical grooming behaviour between animals. Interestingly, the Hucul horse breed was close to extinction after II World War but it was saved thanks to breeding programs, which relied in part on inbreeding; this might has, in fact, result in higher susceptibility to infections to many pathogens, including viruses [55], what can also explain such high prevalence of EFVeca in Hucul horses. Alternatively, the comparably small starting herd might have had a high EFVeca prevalence which is still maintained. The high percentage of EFVeca seropositive horses in purebred group may also be explained by the close contact between animals in big shared stables and during the outdoor activity, while trade in horses between studs may favour the spread of infections. In contrary, livestock horses as well as the group of young Konik horses enrolled in this study are kept in small isolated groups, limiting the possibility of the EFVeca infection.

In many hosts where FVs are prevalent, serum antibodies as well as neutralizing antibodies against FV proteins have been highly correlated with FV infections [22,56]. Beside this, the presence of integrated proviral FV DNA is an independent proof of a new or persistent infection [54]. Our PCR results, which showed the presence of EFVeca DNA in almost 35% (85/248) of tested horses, may indicate that the infection rate of EFVeca in horses was a bit lower than might be suggested by the serological data or that both tests have different sensitivities and specificities. We can assume, that some cases of seropositivity to EFVeca and negative PCR results are due to the fact that horses were exposed to EFVeca antigens but were not productively infected, as it was previously suggested by Romen and co-workers for BFVbta [13]. On the other hand, the relatively high number of PCR negative horse samples, in contrast to the results of ELISA may be caused by the very low number of EFVeca DNA copies in the blood cells of infected animals. Similarly, very low number of provirus copies were also observed in other studies on BFVbta [3], FFVfca [54] and SFVs [57]. In our study, we also noted low 1.6% of horses (9/248 overall and 9/85 in the group of EFVeca PCR-positive horses) showing positive PCR results along to sero-negativity by ELISA. We can suppose that these animals were in an early stage of infection when provirus has been already integrated into host genome but animals did not yet seroconvert. Our previous experimental study on calves and sheep inoculated with BFVbta confirmed the existence of serological window of 2–3 weeks before seroconversion [3]. Since all these nine horses originated from stud farms where EFVeca infected individuals were diagnosed either by PCR or ELISA it is very likely that they represent the individuals at very early stage of infection, without the presence of specific antibodies. Another explanation of this phenomenon may be a much tighter latency of EFVeca in comparison to other known foamy viruses showing lack or rare boost of antibodies by bursts of EFVeca gene expression. Finally in one of PCR positive and ELISA negative horses, a nonsense, stop mutation was noted in the *pol* region, what can be the reason of the replication restriction resulting in the lack or very low level of EFVeca specific antibodies. Altogether, these results showed the discrepancies between ELISA and PCR tests which came from complex nature of foamy virus infections and limitations of diagnostic assays. Additional studies using a new set of samples with a longitudinal access to the animals are needed to study whether these discordant data are due to technical or methodological issues or whether they reflect novel facets in the foamy virus—host interactions.

The diagnostic methods developed in this study may be useful for the screening of possible co-infections of EFVeca with other equine viruses. One of the crucial questions in the FV studies is their potential pathogenic role in infected animals [9,58]. In contrast to defined other retroviruses which are involved in many forms of neoplasia and immunodeficiency syndromes (bovine leukemia virus, feline leukemia virus, feline immunodeficiency virus, small ruminant lentiviruses), FVs are, so far, not linked to specific disease or any clinical signs [59,60,61]; however a fundamental unanswered questions for veterinary science regarding FVs is the extent to which FV infections can be implicated in multifactorial diseases/infections of animals. For example, although no specific pathology has been linked to FFVfca, the recurrent identification of FFVfca in FIV-infected cats raised the possibility of a co-factor role of FFVfca in the onset of feline acquired immunodeficiency [44,62]. Similarly, it has been suggested that simian foamy viruses (SFVs), in their natural hosts or upon zoonotic acquisition by humans, may contribute to disease caused by other retroviruses, like simian immunodeficiency virus [63], or human immunodeficiency virus [64,65]. There are also some data suggesting the positive effect of simian T-leukemia virus type 1 on the replication and transmission of SFV [66] and epidemiological association of BFVbta and bovine leukemia virus and bovine immunodeficiency virus infections in cattle [17,39]. Our study showed possible associations between infections with EFVeca and equine arteritis virus (EAV) (Table 3). Similarly to EFVeca, equine arteritis virus causes persistent infections and is wide-spread in horse population with the 30–60% seroprevalence, which increases with the age of horses [67]. Beside the main venereal routes of transmission EAV can be shed laterally by persistently infected individuals to other horses sharing the same stables through contaminated bedding or fomites as well as the respiratory route [68]. Therefore the association between infection with EFVeca and EAV can simply result from the co-existence of both viruses sharing similar routes of infection but one cannot exclude that both infections somehow influence one another. Either way, it needs to be confirmed by testing of larger and clinically better defined horse cohorts.

In the study presented here, new methods have been developed that can be used for the diagnosis of EFVeca infections in horses, and also confirmed EFVeca infections in horses from several stud farms and representing different breeds in Poland; however, large scale serological and molecular studies with detailed long-term epidemiological observation of EFVeca-incidences will be necessary to extend and confirm our initial findings.

## Figures and Tables

**Table 1:** The prior and posterior medians and lower and upper limits of the posterior equally tailed 95% credible intervals for the sensitivity (Se) and specificity (Sp) of ELISA and western blot (WB).

Parameters	Prior Information	Posterior Information
	Values of Apparent Parameters (Median and the 95% CI)	Values of True Parameters (Median and the 95% CI)
ELISA Se	1.0	0.9731 (0.9172–0.9951)
ELISA Sp	0.935	0.9109 (0.8050–0.9743).
WB Se	0.974 (0.935–0.995)	0.9877 (0.9803–0.9991)
WB Sp	0.944 (0.880–0.983)	0.9953 (0.9903–0.9998)

**Table 2:** Summary of EFVeca Gag ELISA and PCR results in the groups of horses determined by their origin.

Samples Origin	Number of Tested Horses	ELISA Results		PCR Results	
		Negative	Positive	Negative	Positive
Hucul horses	58	16 (27.6%)	42 (72.4%)	31 (55.4%)	27 (46.6%)
The Konik	25	21 (84%)	4 (16%)	25 (100%)	0 (0%)
Livestock horses	66	51 (77.3%)	15 (22.7%)	57 (86.4%)	9 (13.6%)
Saddle horses	17	6 (35.3%)	11 (64.7%)	13 (76.5%)	4 (23.5%)
Purebred horses	82	19 (23.2%)	63 (76.8%)	37 (45.1%)	45 (54.9%)
Total	248	113 (45.6%)	135 (55.6%)	163 (65.7%)	85 (34.3%)

**Table 3:** Summary of ELISA and PCR results.

	PCR (+)	PCR (−)	Total
ELISA (+)	76	59	135
ELISA (−)	9	104	113
Total	85	163	248

**Table 4:** Association between seropositivity to EFVeca and other equine viruses in tested horse samples; *p*-values less than 0.05 were assumed to be statistically significant.

	EIV A1		EIV A2		EAV		EHV-1	
	+	−	+	−	+	−	+	−
EFVeca +	54	6	54	6	52	8	59	1
EFVeca −	19	2	18	3	10	11	19	2
Total	81		81		81		81	
Chi^2^ Test	chi^2^ = 0.004 *p* = 0.9498		chi^2^ = 0.289 *p* = 0.5907		chi^2^ = 13.21 *p* = 0.0003 *		chi^2^ = 2.693 *p* = 0.1008	

*—statistically significant.

## Data Availability

Not applicable.
